# Non-rigorous versus rigorous home confinement differently impacts mental health, quality of life and behaviors. Which one was better? A cross-sectional study with older Brazilian adults during covid-19 first wave

**DOI:** 10.1186/s13690-023-01106-2

**Published:** 2023-06-14

**Authors:** Lucimere Bohn, Pedro Pugliesi Abdalla, Euripedes Barsanulfo Gonçalves Gomide, Leonardo Santos Lopes da Silva, André Pereira dos Santos

**Affiliations:** 1 Faculty of Psychology, Education and Sport, Lusofona University, Porto, Portugal; 2 Faculty of Sport, University of Porto, Porto, Portugal; 3 Research Center in Physical Activity, Health and Leisure (CIAFEL) and Laboratory for Integrative and Translational Research in Population Health (ITR), Porto, Portugal; 4 Study and Research Group in Anthropometry, Training, and Sport (GEPEATE), School of Physical Education and Sport of Ribeirão Preto, University of São Paulo, Ribeirão Preto, SP Brazil; 5 Physical Education Course, Claretiano - Centro Universitário, Batatais, SP Brazil; 6 College of Nursing of Ribeirão Preto, University of São Paulo, Ribeirão Preto, Brazil; 7 School of Physical Education and Sport of Ribeirão Preto, University of São Paulo, Ribeirão Preto, Brazil; 8 Human Exposome and Infectious Diseases Network (HEID), Ribeirão Preto, Brazil

**Keywords:** Public health, Covid-19, Depression, Psychological well-being

## Abstract

**Background:**

The implementation of social distancing measures during covid-19 influenced health outcomes and population´s behaviors, and its rigidity was very different across countries. We aimed to verify the association between the rigidity of social distancing measures of covid-19 first wave with depression symptoms, quality of life and sleep quality in older adults.

**Methods:**

This is a cross-sectional study including 1023 older adults (90% women; 67.68 ± 5.92 years old) of a community-based program in Fortaleza (Brazil). Dependent variables (depression symptoms, sleep quality, and quality of life) were measured through phone calls along June 2020, during the first covid-19 wave. Confinement rigidity (non-rigorous and rigorous) was considered as independent variable. Sociodemographic characteristics (sex, marital status, scholarity, and ethnicity), number of health conditions, nutritional status, movement behavior (physical activity and sitting time), technological skills, and pet ownership were considered as confounding variables. A binomial logistic regression (odds ratio [OR]) was performed to verify the association of confinement rigidity and depression symptoms, sleep quality, and quality of life, adjusted by confounding variables.

**Results:**

Older adults who adopted a less rigid lockdown had a higher frequency of depression symptoms, worse perception of quality of life, and bad sleep quality (p < 0.001). Confinement rigidity was able to explain the probability of depression symptoms occurrence (OR: 2.067 [95% CI: 1.531–2.791]; p < 0.001), worse quality of life (OR: 1.488 [95% CI: 1.139–1.944]; p < 0.05), and bad sleep quality (OR: 1.839 [95% CI: 1.412–2.395]; p < 0.001). Even adjusted by confounding variables, confinement rigidity was able to explain the poor outcomes analyzed in older adults.

**Conclusion:**

Our findings showed that less rigid lockdown was associated with a superior frequency of depression symptoms, worse sleep quality, and lower perception of quality of life in older adults. Therefore, our study could improve comprehension regarding the impact of social distancing measures rigidity in health-related conditions and in the context of covid-19 and other similar pandemic situations.

**Supplementary Information:**

The online version contains supplementary material available at 10.1186/s13690-023-01106-2.

**Table:** 

**Text box 1. Contributions to the literature**
1 – In Brazil, the management of the covid-19 pandemic was carried out in an instructive way, allowing each citizen to opt for a more rigid or less rigid modality.
2 – A more rigid home confinement amongst older Brazilian adults seems to be associated with better mental health indexes (symptoms of depression), perceived quality of life, and sleep quality.
3 - Behavioral and mental problems triggered by the covid-19 outbreak seem to have a milder effect on the daily lives of older Brazilian adults who have adhered to a more rigid home confinement in comparison with a less rigid modality.

## Introduction

The implementation of social distancing measures during covid-19 influenced health-related conditions [1], quality of life [2] and population´s behaviors [3] Nonetheless, the rigidity of social distancing measures was quite variable across countries [4], with some exhibiting a high and others a very low community mobility index during the outbreak first wave [4]. For instance, between April and October of 2020, Brazil had the highest mobility index amongst all South American countries [5]. There is still a lack of studies that compare the effect of the rigidity of social distancing measures on mental health and behavioral outcomes between populations, which is important to help governments to better design public measures for eventual new health crises. For instance, more rigorous social distancing measures have abruptly reduced virus spread, hospital pressure and total deaths [4], but those also impacted on mental health, quality of life and sleep behavior across populations [6]. Conversely, less rigid social distancing measures did not slow down the virus activity, but could also trigger many stressors amongst populations, especially those living in low and middle income countries due to economic constraints [7].

Covid-19 has exacerbated mental stressors amongst populations with an expected increase of 25% in the anxiety and depression prevalence worldwide [8]. Overall, geriatric populations are more likely to suffer from depression symptoms in comparison to other ages [9]. Available data showed an increase from 20% [10] to 30% in the prevalence of depression symptoms amongst older adults as a consequence of the covid-19 first wave [11, 12]. The fear of suffer severe covid-19 infection [13], the interpersonal social support reduction (and even abolishment) due to social distancing measures [14], and financial constraints [15] are factors that explained the increased depression prevalence in older adults during covid-19 first wave [15]. Furthermore, the whole outbreak scenario promoted stressors and lifestyle behaviors disruptions, prejudicing sleep quality and quality of life [8, 16], exacerbating even more anxiety, discomfort, and pain [17]. Moreover, sleep quality elucidated the poorer quality of life during pandemic [18].

Notwithstanding, the impact of rigidity of social distancing measures has not yet been associated with health-related conditions (depression symptoms, sleep quality, and quality of life) in older adults. This gap remains in the current literature.

Thus, this study aimed to verify the association between the rigidity of social distancing measures of covid-19 first wave with depression symptoms, quality of life and sleep quality in older adults. This study could improve comprehension regarding the impact of social distancing measures rigidity in health-related conditions and fornishes subsidies to counteract future pandemic situations.

## Methods

This is a cross-sectional study based on a socioeconomically deprived sample from Fortaleza, Ceará, Brazil. Participants were recruited from the community-based program “Fortaleza Cidade Amiga do Idoso”. Ceará is a northeast Brazilian state with a human development index of 0.735 [19].

To be included, participants should have at least 60 years old and be attending the program in 2019–2020. Data was collected while Ceará citizens were under lockdown lasting, on average, 11.64 ± 2.3 weeks because of the covid-19 outbreak.

Data collection was conducted via telephone interviews during June 2020. A maximum of six telephone attempts (on different days and times) were made to each participant. Data was entered into a Google Form database, which was checked for missing data and typing errors. Participants’ phone numbers were yielded by the Núcleo de Produções Culturais e Esportivas” (NUPROCE) secretariat, the responsible entity for the program, after approval of the ethics board. Verbal consent was provided before any data collection. All procedures were in accordance with the Helsinki Declaration. Further program details and research procedures are elsewhere [18]. This manuscript followed the guidelines from The Strengthening the Reporting of Observational Studies in Epidemiology (STROBE).

## Measurements

### Sociodemographic

Sociodemographic information, such as sex, age, marital status, scholarity, and ethnicity were obtained with open-answer questions. Sociodemographic data were sex (women (code: 0), men (code:1)), marital status (single, divorced and wisdom (code: 0), married or in a relationship (code: 1)), scholarity (less than 6 (code 0), 6 to 12 (code 1) and 12 or more (code 2)), and ethnicity (White (code: 0), Pardo (code 1), Other Ethnicity (Black, Yellow or Indigenous) (code: 2)). The classification of participant’s ethnicity followed the categories used by the Brazilian Institute of Geography and Statistics [20]. Participants’ retirement condition also was inquired as yes (code: 1) or no (code: 0).

### Health conditions

Participants were inquired regarding the presence of health conditions (hypertension, dyslipidemia, diabetes, established cardiovascular diseases, cancer, respiratory diseases and osteoarticular conditions). Answers were compiled as yes (code: 1) or no (code: 0).

The number of above mentioned chronic conditions was computed as the sum of individual diseases (continuous variable).

### Depression symptoms

The validated Brazilian version of the Geriatric Depression Scale–Short Form (GDS-15) [21] was used to ascertain the presence of depression symptoms. The questionnaire is composed of 15 “yes” (code: 1) or “no” (code: 0) questions regarding older adults’ moods during the previous week. The final score is the sum of the 15 questions with superior continuous scores indicating a worse depressive state. GDS-15 results lower than 5 indicate no depression symptoms (code: 0), while GDS-15 ≥ 5 indicates the presence of depression symptoms (code: 1). The test-retest Kappa (K) of the GDS-15 Brazilian version was 0.04 < K < 0.49, and the Spearman’s rho = 0.86 (p < 0.001) [21].

### Quality of life

The adapted Brazilian version of the EuroQol-5D (EQ-5D) questionnaire [22] was used to assess participants’ QoL perception. During the interview, older adults were instructed to report a number between 0 (“the worst QoL you can imagine”) and 100 (“the best QoL you can imagine”) representing their QoL during the lockdown. Results were dichotomized by percentile 50 (P50), which equaled to 70 points (Low QoL: under P50 (code: 1); High QoL: ≥ P50 (code: 0).

### Sleep quality

Sleep quality was assessed with the Brazilian version [23] of the Pittsburgh Sleep Quality Index (PSQI) questionnaire [24], which gauges sleep quality and disturbances from the past month period. The 19-questions from the questionnaire are divided in seven components (subjective sleep quality: C1, sleep latency: C2, sleep duration: C3, habitual sleep efficiency: C4, sleep disturbances: C5, use of sleeping medication: C6 and daytime dysfunction: C7). Each question is scored from 0 to 3 points, and the sum of all individual scores provides the final sleep global score. In terms of classification, good sleep quality (code: 0) is related to 0–5 points, and bad sleep quality (code: 1), to 6–21 points. The computation process of the above mentioned components is described elsewhere in the appendix section [24].

### Nutritional status

Older adults self-reported their body weight (in kg) and height (in meters), and, subsequently, their body mass index (BMI; weight (kg) divided by squared height (m)) was calculated and classified according to the Lipschitz classification as underweight (< 22.00 kg/m^2^), normal (22.00–27.00 kg/m^2^ ) or overweight (> 27.00 kg/m^2^) [25].

### Movement behaviors

#### Physical activity

Habitual physical activity was estimated with the Brazilian validated short version of the International Physical Activity Questionnaire (SV-IPAQ) [26, 27]. Participants provided information about weekly frequency and daily duration they spent in light, moderate, and vigorous physical activities during the past 7 days. Moderate-to-vigorous physical activity was computed following the SV-IPAQ guidelines [26, 27]. Afterwards, participants´ moderate-to-vigorous physical activity was classified according to physical activity recommendations as (non-compliant: <150 min per week of moderate-to-vigorous physical activity; compliant: ≥150 min per week of moderate-to-vigorous physical activity) [28].

#### Daily sitting time

Participants were asked to provide information on how many hours they spent in the sitting position in a normal week and weekend days as stated in the IPAQ-SV [26]. A whole week’s mean of sitting time was computed as ((week sitting time week day x 5 days) + (weekend sitting time week day x 2)) / 7. Further than the continuous variable, daily sitting time of participants were classified as < 8 h/ day of sitting time or ≥8 h/ day of sitting time. [29].

### 24-hour movement guidelines

Participants were classified according to the 24-Hour Movement Guidelines [29]. In brief, participants were classified accordingly daily sitting time (< 8 h/ day of sitting time or ≥ 8 h/ day of sitting time), moderate-to-vigorous physical activity (< 30 min/day or ≥ 30 min/day of moderate-to-vigorous physical activity) and sleep duration (7 to 8 h per day of good-quality sleep) [29]. To be classified as compliant with 24-hour movement guidelines, older adults should have < 8 h/ day of sitting time, ≥ 30 min/day of moderate-to-vigorous physical activity and 7 to 8 h per day of good-quality sleep simultaneously.

### Technological skills and pet ownership

The ability that older adults had to do video-calls and use WhatsApp® in smartphone and pet possession (cats, dogs, and birds) were obtained with “yes” (code: 1) and “not” (code: 0) answers.

### Lockdown rigidity classification

Participants were binary classified according to the rigidity of social distancing measures answering two specific questions. The first, “how many times per week did you leave your home during the lockdown period?” (0 to 2, or 3 or more, answering options). The second, “for how many months did you practice the lockdown?” (until 3 months, or more than 3 months). Participants who leave your home until 2 times per week and practice lockdown more than 3 months were classified as Rigid Lockdown (code: 0). Conversely, those participants who left their homes > 2 times per week and practiced less than 3 months of lockdown were classified as Less Rigid Lockdown (code: 1).

### Statistical procedures

Sample was characterized using absolute frequency, central tendency (mean, median) and dispersion measures (standard deviation and interquartile range). Rigid Lockdown *versus* Less Rigid Lockdown comparisons were performed using both parametric (independent t-test and chi-square (χ^2^) test) and non-parametric tests. Three binomial logistic regressions analysis were used in two steps to explain the binary sleep quality (1 = bad; 0 = good)), QoL (1 = low; 0 = high), and depression symptoms (1 = yes; 0 = no) according to lockdown rigidity (1 = non-rigorous; 0 = rigorous) and sociodemographic data (age (as continuous), sex (female = 0; male = 1), educational level (less than 6 years (ref) = 0; 6 to 12 years = 1; more than 12 years = 3), ethnicity (white (ref) = 0; pardo = 1; other ethnicity = 2), marital status (0 = single, divorced or widowed; 1 = married or common-law marriage), and retirement (0 = no; 1 = yes). The second step was built considering the previous one plus body mass index (0 = normal weight; 1 = overweight and obesity), ability to perform video calls (0 = no; 1 = yes), ability to use WhatsApp® (0 = no; 1 = yes), dog (0 = no; 1 = yes), cat (0 = no; 1 = yes), and bird ownership (0 = no; 1 = yes), number of health conditions (as continuous), compliance with physical activity guidelines (1 = yes; 0 = no), and daily sitting time (as continuous). For each dependent variable, only the significant independent predictors are shown. Models’ significance were verified using the chi-square (χ^2^) test. Significances for each independent variable were analyzed by OR. All procedures were performed using SPSS, version 26 (IBM, Chicago, USA), and *p*-values < 0.05 were considered statistically significant.

## Results

In total, 1,453 older adults were registered in the program in 2019–2020. Of those, 107 had inaccurate phone numbers, 197 did not respond to calls, 11 refused to participate. The final sample comprises 1123 participants (Women: 90%; mean age: 67.68 ± 5.92 years old). Table 1 shows the descriptive statistics according to social distancing measures rigidity (Rigid Lockdown (44.4%); Less Rigid Lockdown (55.6%). Compared to older adults who followed the Less Rigid Lockdown, those under Rigid Lockdown were significantly older (p < 0.001) and with a higher frequency of retirees (p = 0.012). In terms of health conditions, participants of the Less Rigid Lockdown group had a superior frequency of diabetes, cancer, respiratory diseases, and depression symptoms (p < 0.05 for all comparisons). In addition, the Less Rigid Lockdown group had a lower perception of quality of life (p < 0.001) and a superior frequency of bad sleepers in comparison with participants from the Rigid Lockdown group (47.5 vs. 61.4%; p < 0.001, respectively). In terms of movement behaviors, between groups comparisons showed that the Less Rigid Lockdown group exhibits a trend to have less moderate to vigorous physical activity than the Rigid Lockdown group (p = 0.05). In terms of technological skills, older adults from the Less Rigid Lockdown group had a superior frequency in terms of ability to use WhatsApp® in smartphones.

**Table 1:** Descriptive and between groups comparison statistics of Brazilian older adults from Fortaleza, Ceará, Brazil submitted to a home-confinement period during the covid-19 first wave, cross-sectional analysis

Variables	Rigid Lockdown (n: 499)	Less Rigid Lockdown (n:624)	Between group comparisons
**Sociodemographic factors**			
Age, years ± sd	68.96 ± 6.26	66.67 ± 5.43	t (990.930) = 6.468; p < 0.001
Women, n (%)	453 (90.8)	559 (89.6)	χ^2^ (1): 0.447; p = 0.504
**Scholarity, n (%)**			
Less than 6 years	208 (41.7)	225 (36.1)	χ^2^ (2): 6.723; p = 0.035
6 to 12 years	250 (50.1)	360 (57.7)	
12 or more years	41 (8.2)	39 (6.3)	
**Retirement, n (%)**	385 (77.2)	440 (70.5)	χ^2^ (1): 6.274; p = 0.012
**Marital Status, n (%)**			
Single, widowed or divorced	254 (50.9)	324 (51.9)	χ^2^ (2): 0.116; p = 0.390
Married or common-law marriage	245 (49.1)	300 (48.1)	
**Ethnicity, n (%)**			
White	168 (33.8)	223 (35.9)	χ^2^ (2): 3.999; p = 0.135
Pardo	296 (59.6)	373 (60.1)	
Other ethnicity	33 (6.6)	25 (4.0)	
**Health conditions, n (%)**			
Hypertension	315 (63.1)	383 (61.4)	χ^2^ (1): 0.360; p = 0.548
Dyslipidemia	221 (44.3)	283 (45.4)	χ^2^ (1): 0.127; p = 0.722
Diabetes	131 (26.3)	200 (32.1)	χ^2^ (1): 4.485; p = 0.034
Established cardiovascular diseases	45 (9.0)	65 (10.4)	χ^2^ (1): 0.614; p = 0.433
Cancer	14 (2.8)	35 (5.6)	χ^2^ (1): 5.222; p = 0.022
Respiratory diseases	45 (9.0)	100 (16.0)	χ^2^ (1): 12.109; p < 0.001
Osteoarticular conditions	239 (47.9)	296 (47.4)	χ^2^ (1): 0.024; p = 0.878
Number of Health conditions, median [IQR]	2.22 [1.23–3.25]	2.18 [1.15–3.43]	χ^2^ (1) = 0.015; p = 0.904
**Depression Symptoms**			
Depression symptoms, n (%)	114 (22.8)	227 (36.4)	χ^2^ (1): 24.014; p < 0.001
Depression symptoms, median score [IQR]	2.00 [1.00–4.00]	3.00 [2.00–6.00]	χ^2^ (1) = 33.021; p < 0.001
**Quality of Life**			
QoL, mean ± sd	69.83 ± 18.20	62.94 ± 22.57	t (1120.914) = 5.661; p < 0.001
Low QoL (< P50), n (%)	278 (41.2)	396 (58.8)	χ^2^ (1) = 6.940; p = 0.005
High QoL (> P50), n (%)	221 (49.2)	228 (50.8)	
**Sleep quality, n (%)**			
Good	262 (52.5)	241 (38.6)	χ^2^ (1): 21.612; p < 0.001
Bad	237 (47.5)	383 (61.4)	
**Nutritional Status**			
Body mass index, mean kg/m^2^ ± sd	27.02 ± 4.07	27.14 ± 3.95	t (1,099)= -0.520; p = 0.603
Underweight, n (%)	40 (8.2)	35 (5.7)	χ^2^ (2): 2.598; p = 0.273
Normal weight, n (%)	222 (45.4)	284 (46.4)	
Overweight and obesity, n (%)	227 (46.8)	293 (47.9)	
**Movement Behaviors**			
Daily sitting time, mean hours ± sd	6.17 ± 2.62	5.18 ± 2.22	t (1112) = 6.848; p < 0.001
Daily MVPA, median minutes [IQR]	11.43 [0.00–25.71]	8.57 [0.00–21.43]	χ^2^ (1) = 3.837; p = 0.050
Compliant with MVPA guidelines, n (%)	112 (22.4)	122 (19.7)	χ^2^ (1): 1.219; p = 0.270
Meeting 24-h Canadian Guidelines, n (%)	36 (7.2)	32 (5.1)	χ^2^ (1): 2.121; p = 0.145
**Technological skills, n (%)**			
Ability to do video calls	240 (48.1)	317 (50.8)	χ^2^ (1): 0.812; p = 0.368
Ability to use WhatsApp®	325 (65.1)	466 (74.7)	χ^2^ (1): 12.142; p < 0.001
**Pet ownership, n (%)**			
Dog	156 (31.3)	207 (33.2)	χ^2^ (1): 0.463; p = 0.269
Cat	116 (23.2)	137 (22)	χ^2^ (1): 0.265; p = 0.329
Bird	68 (13.6)	102 (16.3)	χ^2^ (1): 1.596; p = 0.207

*Note*: IQR: interquartile range; MVPA: Moderate-to-vigorous physical activity

Table 2 shows the probability of explaining presence of depression symptoms, lower quality of life, and bad sleep quality risk with logistic regressions based on confinement rigidity, sociodemographic variables (age, sex, ethnicity, marital status, retirement, educational level), pet ownership, technological skills, number of health conditions, nutritional status, compliance with physical activity guidelines and daily sitting time. Only those independent predictors that significantly explain the three outcomes are presented in Table 2. Regardless of the outcome, confinement rigidity was able to explain the probability of depression symptoms occurrence (p < 0.001), worse quality of life (p = 0.019) and bad sleep quality (p < 0.001), independently and effectively of confound variables. In regards to depression symptoms, together with lockdown rigidity, some correlates reduced the odds of depression symptoms (12 or more years of education (OR: 0.360; p = 0.003) and compliance with physical activity recommendations (OR: 0.647; p = 0.020)), while others increased it (ethnicity [pardos and other ethnicity]), number of health conditions and ability to use WhatsApp®. The number of health conditions (OR: 1.1380; p < 0.001) and daily sitting time (OR: 1.1092; p = 0.002) augmented the odds of having a poor quality of life. In the case of sleep, further than confinement rigidity, ethnicity different of white (p < 0.05), number of health conditions (p < 0.001), compliance with physical activity guidelines (p = 0.004) and daily sitting time (p = 0.002) have explained the probability of a bad sleep quality pattern.

**Table 2:** Probability of explaining the presence of depression symptoms risk, lower quality of life, and bad sleep quality with logistic regressions by cross-sectional analysis based on confinement rigidity, sociodemographic variables, pet ownership, technological skills, and number of health conditions, nutritional status, and fulfillment of physical activity guidelines. Brazilian older adults’ data from Fortaleza, Ceará, Brazil submitted to a home-confinement period during the covid-19 first wave

Variable		χ^2^	*p*	Wald	OR	*p*	95%CI [LB-SB]
Dependent	Independent						
**Depression Symptoms** **(1 = yes; 0 = no)**	Confinement rigidity (1 = non-rigorous; 0 = rigorous)	134.033	< 0.001	22.474	2.067	< 0.001	1.531–2.791
	Educational level						
	- Less than 6 years (ref)			9.516		0.009	
	− 6–12 years			0.881	0.865	0.348	0.639–1.171
	- More than 12 years			8.965	0.360	0.003	0.184–0.703
	Ethnicity						
	- White (ref)			13.567		0.001	
	- Pardos			5.951	1.461	0.015	1.077–1.982
	- Other ethnicity			8.653	2.456	0.003	1.350–4.469
	Ability to use WhatsApp® (0 = no; 1 = yes)			10.886	2.031	< 0.001	1.333–3.093
	Number of health conditions (continuous)			39.401	1.371	< 0.001	1.243–1.514
	Compliance with physical activity recommendations (1 = yes; 0 = no)			5.417	0.647	0.020	0.448–0.934
**Quality of Life** **(1 = bad; 0 = good)**	Confinement rigidity (1 = non-rigorous; 0 = rigorous)	74.540	< 0.001	8.476	1.488	0.004	1.139–1.944
	Number of health conditions (continuous)			43.789	1.380	< 0.001	1.254–1.518
	Daily sitting time (continuous)			9.721	1.092	0.002	1.033–1-154
**Sleep Quality** **(1 = bad; 0 = good)**	Confinement rigidity (1 = non-rigorous; 0 = rigorous)	91.290	< 0.001	20.427	1.839	< 0.001	1.412–2.395
	Ethnicity						
	- White (ref)			7.065		0.029	
	- Pardos			3.365	1.285	0.067	0.983–1.679
	- Other ethnicity			4.093	1.865	0.043	1.020–3.411
	Number of health conditions (continuous)			32.965	1.312	< 0.001	1.196–1.439
	Fulfilling physical activity recommendations (1 = yes; 0 = no)			8.202	0.637	0.004	0.468–0.867
	Daily sitting Time (continuous)			6.331	0.933	0.012	0.884–0.985

*Note*: For all dependent variables Depression Symptoms (1 = yes; 0 = no), Quality of Life (1 = low; 0 = high) and Sleep Quality (1 = bad; 0 = good), the independent variables age (as continuous), sex (female = 0; male = 1), educational level (less than 6 years (ref) = 0; 6 to 12 years = 1; more than 12 years = 3), ethnicity (white (ref) = 0; pardo = 1; other ethnicity = 2), marital status (0 = single, divorced or widowed; 1 = married or common-law marriage), retirement (0 = no; 1 = yes), nutritional status (0 = normal weight; 1 = overweight and obesity), ability to perform video calls (0 = no; 1 = yes), ability to use WhatsApp® (0 = no; 1 = yes), dog ownership (0 = no; 1 = yes), cat ownership (0 = no; 1 = yes), bird ownership (0 = no; 1 = yes), number of health conditions (as continuous), compliance with physical activity guidelines (1 = yes; 0 = no), and daily sitting time (as continuous) were considered. For each dependent variable, only the significant independent predictors are shown. χ^2^: Chi-square test; OR: odds ratio or Exp(B); CI: confident interval; LB: lower bond; UB: upper bond.

## Discussion

The study verified the association of social distancing measures rigidity from covid-19 with depression symptoms, quality of life and sleep quality in older adults. To the best of our knowledge this is the first study that evaluated the impact of different lockdown rigidity from covid-19 in distinct health related aspects together in older adults. Participants who adopted a less rigid lockdown had a superior frequency of depression symptoms, were more frequently classified as bad sleepers, and had a lower perception of quality of life. Additionally, in our sample, less rigid lockdown increases at least one and a half times, worse outcomes of depression symptoms, sleep quality and quality of life in older adults.

Older adults experienced an expressive reduction in their life-space mobility with the lockdown, influenced by the impact of the covid-19 quarantine [30]. Said that, it was expected that older adults would suffer with lower quality of life perceived perception, poorer sleep quality and increased depression symptoms [31]. However, our findings are appointed to explain these expected phenomena in an association with the confinement rigidity. The confinement rigidity was able to explain the probability of bad sleep quality, worse quality of life and depression symptoms, independently and effectively of confound variables. In this sense, older adults who adopted less rigid lockdowns had a lower perception of quality of life, worse sleep behavior and showed more depression symptoms. A recent meta-analysis showed that different populations respond differently to the psychological stress and poor quality of life generated by the pandemic and its confinement measures [32]. These individual responses can occur because of household income, insecurity about the pandemic context, misinformation about the vaccination, and the numerous deaths [33].

Scrutinizing each finding separately, we highlighted the association of confounding variables (see Table 2) with bad sleep quality, poor quality of life, and augmented depression symptoms. Bad sleep quality patterns were also associated with ethnicity different of white, number of health conditions, compliance with physical activity guidelines, and daily sitting time. The number of health conditions and daily sitting time augmented the odds of having a poor quality of life. Ethnicity (pardos and other ethnicity), number of health conditions and ability to use WhatsApp® increased the odds of depressive symptoms, whereas 12 or more years of education, and compliance with physical activity recommendations reduced this odds. In fact, these confounding variables were also reported in previous studies [34, 35], and the researchers called attention to the behavioral variables (i.e., physical activity, sitting time, and use of electronic media). The maintenance of physical health was impaired by social isolation, and it becomes clear with the decrease in physical activity (consequent increase of sitting time), an important protective factor for older adults [35]. By the other hand, the use of electronic media (e.g., Whatsapp®) had not been associated with greater depressive symptoms [36], and our study showed the inverse. We argue that the specificity of our sample showed worsening of depression symptoms with the ability to use WhatsApp®, suggesting psychological distress with the scenario that was unclear during the covid-19 pandemic.

Other findings of our study must be highlighted and discussed with the previous literature. Compared to older adults who followed the less rigid lockdown, those under rigid lockdown were significantly older and with a higher frequency of retirees. This can occur due to the economic consequences of the pandemic, contributing to higher rates of monthly income insecurity among households and causing some older adults to adopt a less rigid lockdown regime [33]. Moreover, older adults who adopted less rigid lockdowns had a superior frequency of diabetes, cancer, respiratory diseases, insufficient moderate-to-vigorous physical activity, and ability to use WhatsApp® in smartphones. Diabetes, cancer, and insufficient physical activity are considered risk factors for severity of covid-19, while the use of mobile phones is associated with loneliness perception [37, 38]. Therefore, our findings assigned to a risk scenario regarding the older adults who adopted less rigid lockdown in addition to the factors mentioned in previous paragraphs (see Table 2).

The strengths of the study include the large number of older adults. In addition, the data collection was acquired by well-trained researchers who clearly and precisely explained each question, avoiding misunderstandings as it might happen when older adults fulfill online questionnaires. Data was based on a convenience sample from only one geographical area (Fortaleza, Ceará). Depression and sleep quality have other determinants (i.e., number of positive social contacts, polypharmacy, financial constraints, amongst many others) that were not considered in this analysis. The classification about the rigidity lockdown was based on national-level policies and decisions (head of state). However, in Brazil, the main decision of accomplishing with rigidity lockdown was more individual level. In this sense, our findings need to be interpreted with caution, considering the variability and particularity of each individual, country, and adopted measures during covid-19 pandemic [39]. In addition, one important weakness of the study is the lack of control for many other confounding factors that potentially could influence our regression models and thus represent a bias of risk.

The study results are useful for policymakers to design strategies to counteract depression symptoms, sleep quality, and quality of life in older adults, during situations needing social distancing measures, which could be more or less rigid, according to public policies and individual contexts.

## Conclusion

The less rigid lockdown was associated with a superior frequency of depression symptoms, worse sleep quality, and lower perception of quality of life in older adults. This study could improve comprehension regarding the impact of social distancing measures rigidity in health-related conditions and in the context of covid-19 and other similar pandemic situations.

## Electronic supplementary material

Below is the link to the electronic supplementary material.

Supplementary Material 1

## Data Availability

Not applicable.
