# Supplementary material for: Non-rigorous versus rigorous home confinement differently impacts mental health, quality of life and behaviors. Which one was better? A cross-sectional study with older Brazilian adults during covid-19 first wave
Source: Arch Public Health. 2023 Jun 14;81:105. doi: 10.1186/s13690-023-01106-2 (PMC10265552; doi:10.1186/s13690-023-01106-2)
Supplement: Supplementary file 1 — Supplementary Material 1 [file 13690_2023_1106_MOESM1_ESM.docx]

**Letter to Editor – Aims and Scope statement**

Brazil, January 05th 2023.

Dear Editors-in-Chief of the *Archives of Public Health,*

*Olivier Bruyère, PhD*

*Herman Van Oyen, PhD*

We are pleased to submit the manuscript entitled: **“Non-rigorous versus rigorous home confinement differently impacts mental health, quality of life and older adults' behaviors. Which one was better?** **a cross-sectional study”** to the *Archives of Public Health*.

Covid-19 has exacerbated mental stressors amongst populations with an expected increase of 25% in the anxiety and depression prevalence worldwide [1]. Overall, geriatric populations are more likely to suffer from depression symptoms in comparison to other ages [2]. Available data showed an increase from 20% [3] to 30% in the prevalence of depression symptoms amongst older adults as a consequence of the covid-19 first wave [4]. The fear of suffer severe covid-19 infection, the interpersonal social support reduction (and even abolishment) due to social distancing measures, and financial constraints are factors that explained the increased depression prevalence in older adults during covid-19 first wave [5]. Furthermore, the whole outbreak scenario promoted stressors and lifestyle behaviors disruptions, prejudicing sleep quality and quality of life, exacerbating even more anxiety, discomfort, and pain [1,6]. Moreover, sleep quality elucidated the poorer quality of life during pandemic.

The study verified the association of social distancing measures rigidity from covid-19 with depression symptoms, quality of life and sleep quality in older adults. To the best of our knowledge this is the first study that evaluated the impact of different lockdown rigidity from covid-19 in distinct health related aspects together in older adults. Participants who adopted a less rigid lockdown had a superior frequency of depression symptoms, were more frequently classified as bad sleepers, and had a lower perception of quality of life. Additionally, in our sample, less rigid lockdown increases at least one and a half times, worse outcomes of depression symptoms, sleep quality and quality of life in older adults.

**References**

1 - WHO. (2022). COVID-19 pandemic triggers 25% increase in prevalence of anxiety and depression worldwide. Retrieved 20 oct from https://www.who.int/news/item/02-03-2022-covid-19-pandemic-triggers-25-increase-in-prevalence-of-anxiety-and-depression-worldwide

2 - Arthur, A., Savva, G. M., Barnes, L. E., Borjian-Boroojeny, A., Dening, T., Jagger, C., Matthews, F. E., Robinson, L., & Brayne, C. (2020). Changing prevalence and treatment of depression among older people over two decades. Br J Psychiatry, 216(1), 49-54. https://doi.org/10.1192/bjp.2019.193

3 - Volkert, J., Schulz, H., Härter, M., Wlodarczyk, O., & Andreas, S. (2013). The prevalence of mental disorders in older people in Western countries - a meta-analysis. Ageing Res Rev, 12(1), 339-353. https://doi.org/10.1016/j.arr.2012.09.004

4 – Herrera, M. S., Elgueta, R., Fernández, M. B., Giacoman, C., Leal, D., Marshall, P., Rubio, M., & Bustamante, F. (2021). A longitudinal study monitoring the quality of life in a national cohort of older adults in Chile before and during the COVID-19 outbreak. BMC Geriatr, 21(1), 143. https://doi.org/10.1186/s12877-021-02110-3

5 - Pinto, J., van Zeller, M., Amorim, P., Pimentel, A., Dantas, P., Eusébio, E., Neves, A., Pipa, J., Santa Clara, E., Santiago, T., Viana, P., & Drummond, M. (2020). Sleep quality in times of Covid-19 pandemic. Sleep Med, 74, 81-85. https://doi.org/https://doi.org/10.1016/j.sleep.2020.07.012

6 - Abdalla, P. P., Neto, S. d. Q. E., de Souza Lage, S. A. C., Gomes, S., de Freitas, B. M. d. D., Pedro-Costa, S., Machado, R. L. D., Oliveira, J., Mota, J., & Bohn, L. (2022). Sleep Quality and Quality of Life Among Older Adults During COVID-19 Pandemic: A Cross-Sectional Study. Current Aging Science, 15(2), 186-196. https://doi.org/http://dx.doi.org/10.2174/1874609815666220304195647

**Aims and Scope statement**

**1. What is known?**

The impact of rigidity of social distancing measures due to covid-19 pandemic has not yet been associated with health-related conditions (depression symptoms, sleep quality, and quality of life) in older adults.

**2. What does the study adds**

The less rigid lockdown was associated with a superior frequency of depression symptoms, worse sleep quality, and lower perception of quality of life in older adults. This study could improve comprehension regarding the impact of social distancing measures rigidity in health-related conditions and in the context of covid-19 and other similar pandemic situations.

**3. What are implications for clinical practice, public health and / or research?**

The study results are useful for policymakers to design strategies to counteract depression symptoms, sleep quality, and quality of life in older adults, during situations needing social distancing measures, which could be more or less rigid, according to public policies and individual contexts.

Thank you for the opportunity to submit our work in this great journal!
Kind regards,

Authors,
